# Case Report: Sapien 3 Transcatheter Heart Valve Embolization: Cause, Management, and Redo

**DOI:** 10.3389/fcvm.2021.774047

**Published:** 2021-11-02

**Authors:** Mi Chen, Barbara E. Stähli, Jonathan M. Michel, Miriam Brinkert, Felix C. Tanner, Albert Markus Kasel

**Affiliations:** ^1^University Heart Center, University Hospital Zurich, Zurich, Switzerland; ^2^Department of Cardiac Surgery, Beijing Anzhen Hospital, Capital Medical University, Beijing, China; ^3^Department of Cardiology, Kantonsspital Aarau, Aarau, Switzerland

**Keywords:** TAVI – transcatheter aortic valve implantation, embolization, transcatheter heart valve (THV), Sapien 3, aortic stenosis

## Abstract

The transcatheter heart valve (THV) embolization is a rare but challenging complication in transcatheter aortic valve implantation (TAVI). We report the case of an 81-year-old man with Sapien 3 embolization caused by interrupted rapid pacing. In this setting, we describe the embolized THV management and the technique of the second Sapien 3 implantation.

## Patient History

An 81-year-old man with hypertension, permanent atrial fibrillation, and chronic renal disease presented to the cardiology department complaining of syncope and dyspnea. Transthoracic echocardiography (TTE) showed severe paradoxical low-flow, low-gradient aortic stenosis of a tricuspid aortic valve (mean transaortic pressure gradient = 33 mmHg; aortic valve area = 0.96 cm^2^; indexed aortic valve area = 0.49 cm^2^/m^2^; aortic valve V_max_ = 3.66 m/s; left ventricular ejection fraction = 63%) (1). Pre-transcatheter aortic valve implantation (TAVI) computed tomography (CT) demonstrated a large aortic annulus with only minor calcifications (annulus area = 685 mm^2^, area-derived diameter = 29.53 mm). Coronary angiography showed insignificant coronary artery stenosis.

## Management

### Embolization of Sapien 3 THV

The Heart Team decided to proceed with transfemoral TAVI given the patient's age, frailty, and the burden of comorbidities. Based on the annulus dimensions, TAVI with a 29-mm Sapien 3 (Edwards Lifesciences, Irvine, California) balloon-expandable transcatheter heart valve (THV) with 2 ml overfilling was planned.

**Figure 1:**
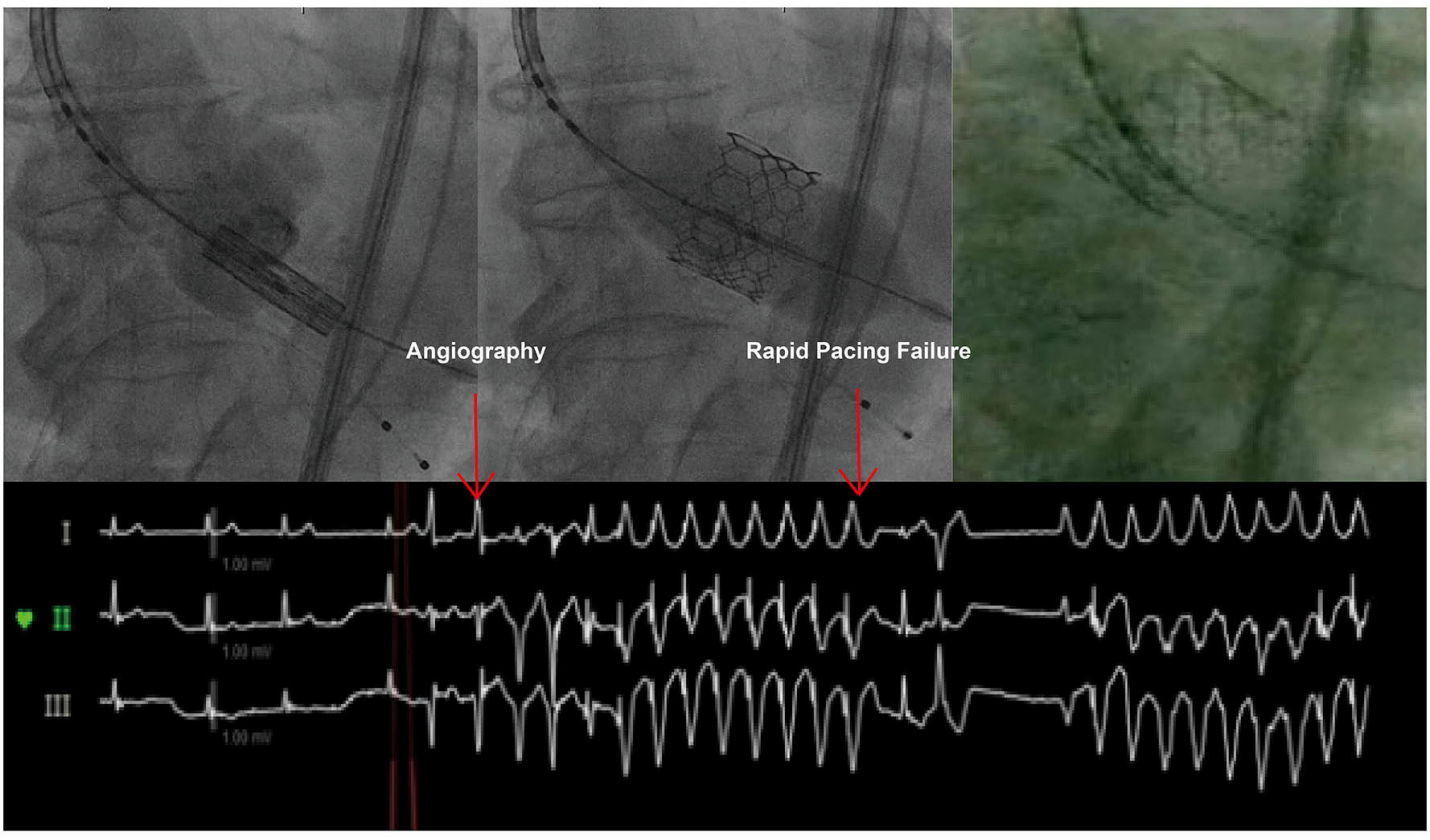
Sapien 3 THV Embolization. Failure of rapid pacing caused Sapien 3 embolization.

Transcatheter aortic valve implantation was performed under conscious sedation in the hybrid operating room. Having obtained vascular access, the THV was advanced and positioned within the aortic annulus. After rapid pacing had started, the accurate placement height of the Sapien 3 THV was checked by angiography. The Sapien 3 THV was inflated gradually to approach the aortic annulus. As soon as the frame of the Sapien 3 THV had touched the hinge points of the annulus, rapid pacing was discontinued accidentally. The Sapien 3 THV popped out into the ascending aorta, followed by immediate deflation of the deploying balloon (Figure 1).

### Management of Sapien 3 THV Embolization

**Figure 2:**
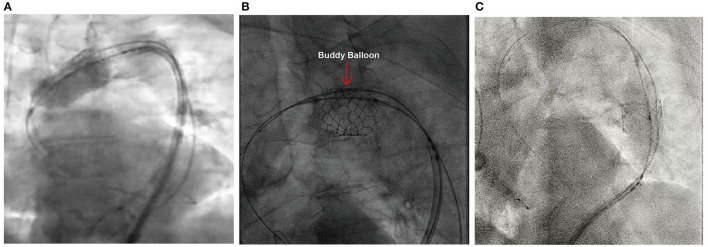
Repositioning of the Embolized Sapien 3 THV. **(A)** Inflation of the deploying balloon to pull the whole system back. **(B)** Compression of the Sapien 3 THV by inflating a “buddy balloon.” **(C)** Anchoring of the embolized Sapien 3 THV into the descending aorta.

First, management consisted of THV repositioning and anchoring, without causing any side branch obstruction. After keeping the stiff wire fixed in the left ventricle (LV) to avoid rotation of the THV, we inflated the deployment balloon and pulled it inside the inflow of the THV, thereby retrieving the whole system gently (Figure 2A). The delivery system with the trapped THV was retracted smoothly but encountered resistance just before the origin of the left subclavian artery. We, therefore, attempted to crimp the Sapien 3 slightly. We used the pigtail catheter, pre-positioned for aortic root angiography, as a “buddy wire” to advance a 10 × 40-mm balloon with a long J-wire (Figure 2B). A parallel access outside of the embolized THV in the arch was established to ensure the further manipulation. Having positioned the balloon adjacent to the Sapien 3 THV, the balloon was inflated and the Sapien 3 THV thereby compressed with counteractive force. The whole delivery system with the THV trapped by the deployment balloon could then be retrieved smoothly into the descending aorta.

The descending aorta was considered as an ideal anchoring position without origins of brachiocephalic arteries and other primary arteries. Sizing of the descending aorta was performed on the pre-TAVI CT. A location with a diameter of 28 mm was considered ideally to anchor without the further use of stenting. Then the Sapien 3 THV anchored in the descending aorta with 2 ml overfilling of the deploying balloon (Figure 2C).

### Deployment of the Second Sapien 3 THV

The second Sapien 3 THV was implanted and delivered through the first Sapien 3 THV. To avoid leaflet damage of the first Sapien 3 THV, the delivery system was half-flexed to pass the first Sapien 3 THV centrally. Balloon alignment of the delivery system was commenced in the ascending aorta with adequate space but more resistance. The second Sapien 3 THV was deployed with 4 ml overfilling, given that the annulus of 29.5 mm is outside of the recommended range (2, 3). “Flare the outflow” technique was performed at the height of the Sapien 3 outflow to achieve safe anchoring and preventing potential valve migration or embolization toward the left ventricle (LV) (4).

### Post-implantation Management

**Figure 3:**
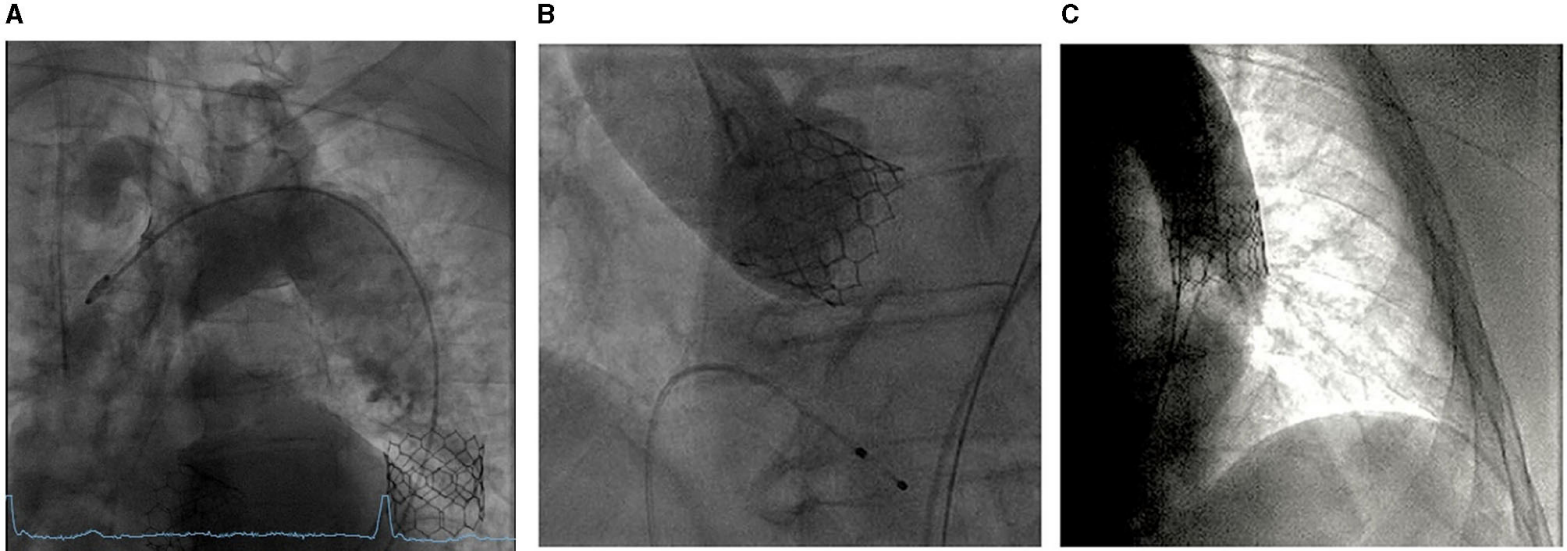
Post-implantation evaluation. **(A)** Aortic root angiography for the evaluation of the second Sapien 3 THV. **(B)** Descending aorta angiography for the evaluation of the anchored Sapien 3 THV. **(C)** Aortic arch angiography for the exclusion of any occlusion of the main arteries.

Post-implantation management consisted of routine THV functional evaluation, ensuring anchoring of the embolized THV and excluding potential occlusion of main arteries. Invasive pressure assessment showed no transvalvular pressure gradient of the second THV. Aortic root angiography in functional projection showed an excellent result without any paravalvular leak (Figure 3A). The embolized Sapien 3 THV has inflated again with the second delivery system to ensure optimal anchoring. Angiography of the aortic arch and descending aorta was performed to confirm the optimal position of the second Sapien 3 THV and exclude any occlusion of the main arteries or migration of the embolized Sapien 3 THV (Figures 3B,C). The patient was under conscious sedation and in stable hemodynamic conditions throughout the procedure. The duration of the procedure was 85 min.

## Discussion

**Figure 4:**
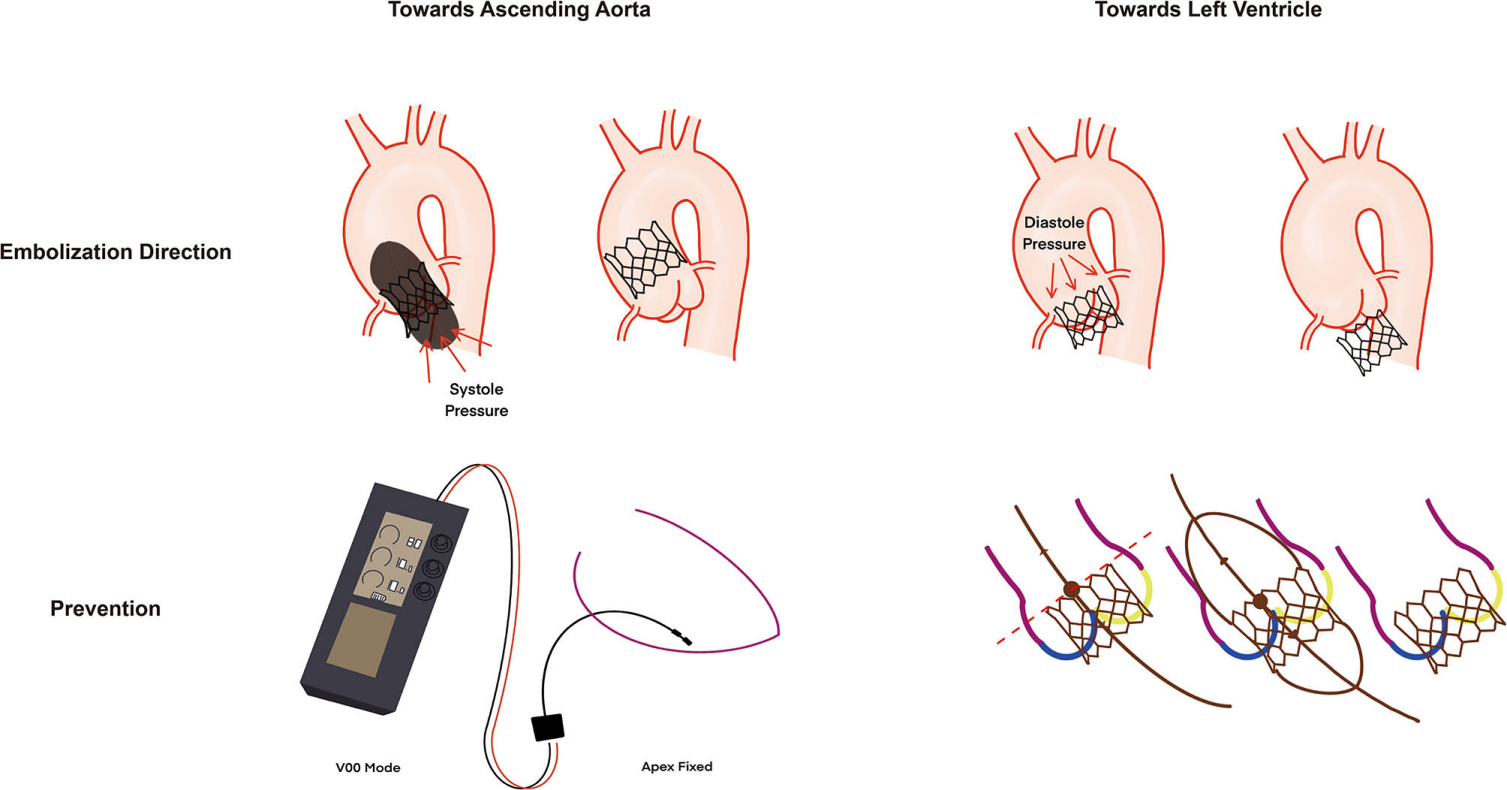
Mechanism of BEV embolization and prevention. BEV can migrate toward ascending aorta (systole pressure caused by rapid pacing failure) or left ventricle (diastole pressure). Adequate assessment of rapid pacing before implantation with V00 mode and fixed apex position and sufficient anchor with the “flare the outflow” technique could enhance the anchor of BEV. BEV, balloon expandable valve.

Transcatheter heart valve embolization is a rare but challenging complication of TAVI, with a reported incidence of about 1% (5). Rapid pacing failure remains one of the main individual causes of THV embolization, along with malpositioning, manipulation, and post-dilatation, with a higher prevalence in balloon-expandable valves (BEV) (5). Transcatheter heart valve embolization occurs toward the LV in case of insufficient anchoring of the valve (aortic pressure pushes the closed valve toward the left ventricle) or toward the aorta when rapid pacing during BEV deployment is interrupted (LV pressure pushes the valve toward the aorta) (Figure 4) (4). In this case, unexpected discontinuation of rapid pacing occurred without any obvious user error, which may be unavoidable. However, an adequate pre-implantation rapid pacing test should be confirmed carefully.

In this case, we summarized the management of an embolized balloon-expandable THV, including repositioning and anchoring of the embolized THV, implantation of the second THV, and post-implantation evaluation. Repositioning of the THV includes retraction of the valve on the balloon-shoulder with an inflated balloon, a snare, or a bioptome, which are, however, both associated with a substantial risk of aortic injury (6). As our first bailout strategy, the local deployment balloon was inflated again for retrieval with minimal invasiveness (embolized Sapien 3 THV is centered on the balloon-shoulder and not tilted). When the inflated THV was then stuck in the aortic arch, we used the second bailout strategy, consisting of a “buddy wire” and the advancement and inflation of a 10 x 40-mm balloon beside the embolized THV to compress the fully deployed valve and to bring it away from the outer curvature of the aortic arch and the outgoing arteries.

**Figure 5:**
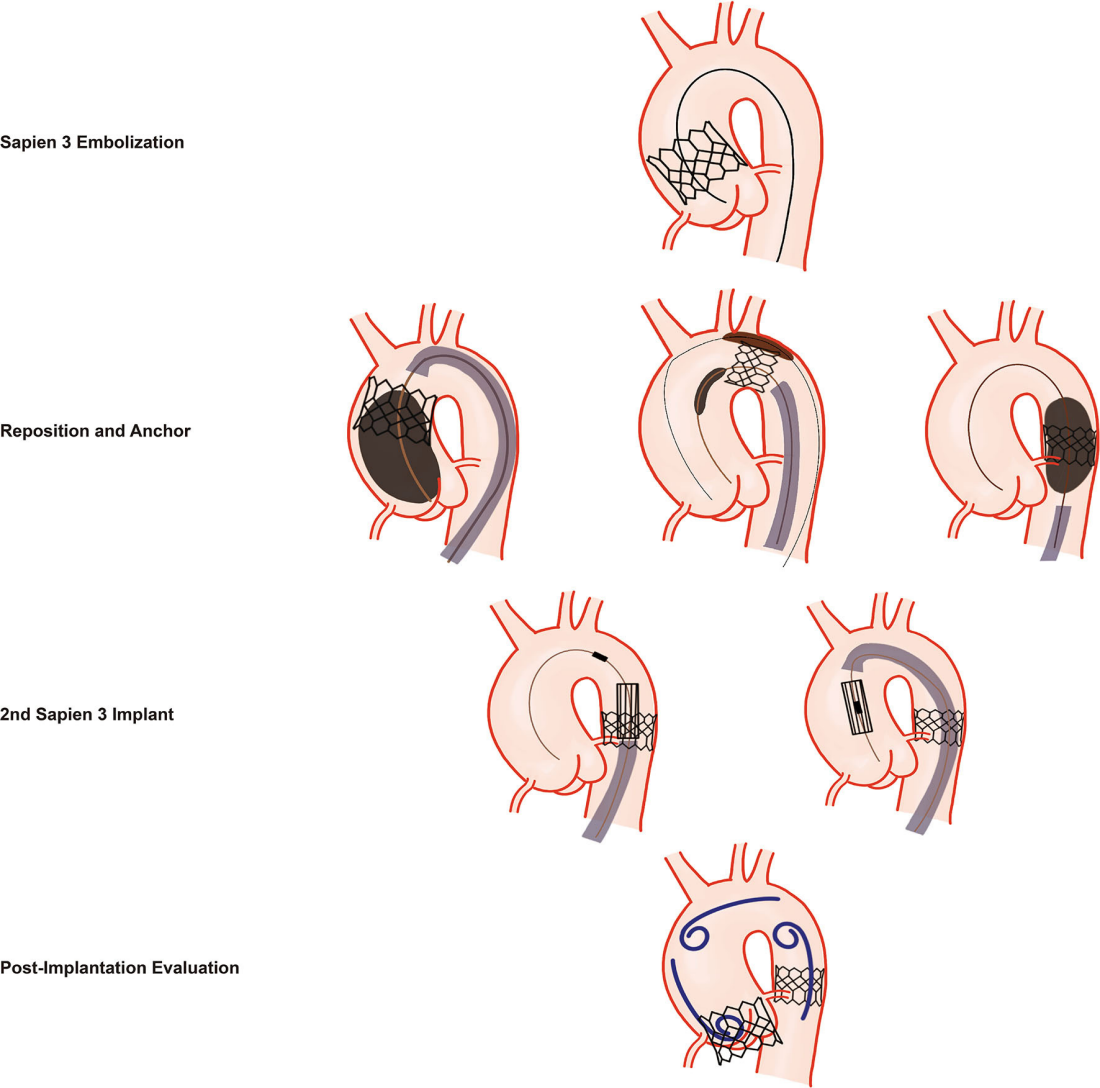
Management of Sapien 3 THV Embolization. Schematic illustration of the management of a Sapien 3 THV embolization. First, repositioning and anchoring of the embolized Sapien 3 THV into the descending aorta with minimally invasive approaches are performed. Second, implantation of the second Sapien 3 THV with maximal centralization of the delivery system when crossing the embolized Sapien 3 THV. Third, angiography of the aortic root, the aortic arch, and the descending aorta is performed to evaluate THV function, patency of the brachiocephalic arteries, and the stability of the embolized valve in the descending aorta.

When embolization has occurred, a well-cooperating Heart Team is crucial to successfully tackle the complication. Overall management is performed by the operators (Figure 5, Supplementary Video 1). The scrub nurse is responsible to prepare and crimp the second THV immediately, and anesthesiologists are in charge of maintaining stable hemodynamics. Mechanical circulation support should be on standby when it is necessary.

## Follow-Up

After the procedure, the patient was sent to the intermediate care unit. He was conscious without neurologic symptoms. Electrocardiography showed atrial fibrillation with bradycardia. Laboratory tests had no positive findings. The preexisting anticoagulation with a non-vitamin K antagonist oral anticoagulant (NOAC) was continued 6 h after the intervention.

## Conclusions

Failure of rapid pacing may cause THV embolization. Overall management of this complication should include repositioning the first THV, modified implantation of the second THV, and an adequate THV evaluation after the second implantation.

## Learning Objectives

- Failure of rapid pacing could cause THV embolization toward the ascending aorta.
- Embolization management includes first THV repositioning, second THV implantation, and careful post-implantation evaluation.
- The Sapien 3 THV could be compressed and navigated successfully through the aortic arch by using a “buddy balloon” and performing a pullback maneuver into the descending aorta on the shoulder of the inflated deployment balloon.

## Data Availability Statement

The raw data supporting the conclusions of this article will be made available by the authors, without undue reservation.

## Author Contributions

MC and AK conceived of the presented idea of the paper. MC wrote the manuscript with support from AK, BS, JM, MB, and FT. MC drew the figures. AK supervised the project. All authors contributed to the article and approved the submitted version.

## Conflict of Interest

AK is a consultant and proctor for Edwards Lifesciences. The remaining authors declare that the research was conducted in the absence of any commercial or financial relationships that could be construed as a potential conflict of interest.

## Publisher's Note

All claims expressed in this article are solely those of the authors and do not necessarily represent those of their affiliated organizations, or those of the publisher, the editors and the reviewers. Any product that may be evaluated in this article, or claim that may be made by its manufacturer, is not guaranteed or endorsed by the publisher.

## Supplementary Material

Supplementary Video 1 Embolized Sapien 3 THV: Cause, management, and redo. THV, transcatheter heart valve.

## Abbreviations

BEV: Balloon Expandable Valve
LV: Left Ventricle
TAVI: Transcatheter Aortic Valve Implantation
THV: Transcatheter Heart Valve.

## References

[1] Writing Committee Members Otto CM Nishimura RA Bonow RO Carabello BA Erwin JP III. 2020 ACC/AHA guideline for the management of patients with valvular heart disease: a report of the American College of Cardiology/American Heart Association Joint Committee on clinical practice guidelines. J Am Coll Cardiol. (2021) 77:e25–197. 10.1016/j.jacc.2020.11.018 33342586

[2] Shivaraju A Kodali S Thilo C Ott I Schunkert H von Scheidt W. Overexpansion of the SAPIEN 3 transcatheter heart valve: a feasibility study. JACC Cardiovasc Interv. (2015) 8:2041–3. 10.1016/j.jcin.2015.10.006 26738676

[3] Tang GHL Zaid S George I Khalique OK Abramowitz Y Maeno Y. Impact of aortic root anatomy and geometry on paravalvular leak in transcatheter aortic valve replacement with extremely large annuli using the edwards SAPIEN 3 valve. JACC Cardiovasc Interv. (2018) 11:1377–87. 10.1016/j.jcin.2018.03.034 29960755

[4] Chen M Michel J Kasel AM. Application of balloon-expandable transcatheter heart valve in bicuspid aortic valve. JACC: Asia. (2021) 1:147–61. 10.1016/j.jacasi.2021.07.012 36338163 PMC9627836

[5] Kim WK Schafer U Tchetche D Nef H Arnold M Avanzas P. Incidence and outcome of peri-procedural transcatheter heart valve embolization and migration: the TRAVEL registry (TranscatheteR HeArt Valve EmboLization and Migration). Eur Heart J. (2019) 40:3156–65. 10.1093/eurheartj/ehz429 31230081

[6] Alkhouli M Sievert H Rihal CS. Device embolization in structural heart interventions: incidence, outcomes, and retrieval techniques. JACC Cardiovasc Interv. (2019) 12:113–26. 10.1016/j.jcin.2018.08.033 30678792

